# Testing Efficacy of Assembly-Free and Alignment-Free Methods for Species Identification Using Genome Skims, with Patellogastropoda as a Test Case

**DOI:** 10.3390/genes13071192

**Published:** 2022-07-02

**Authors:** Tao Xu, Lingfeng Kong, Qi Li

**Affiliations:** 1 Key Laboratory of Mariculture, Ministry of Education, Ocean University of China, 5 Yushan Road, Qingdao 266003, China; xutao9611@163.com (T.X.); qili66@ouc.edu.cn (Q.L.); 2 Laboratory for Marine Fisheries Science and Food Production Processes, Qingdao National Laboratory for Marine Science and Technology, 5 Yushan Road, Qingdao 266003, China

**Keywords:** genome skims, genomic distance, phylogenetic placement, patellogastropoda

## Abstract

Most recently, species identification has leaped from DNA barcoding into shotgun sequencing-based “genome skimming” alternatives. Genome skims have mainly been used to assemble organelle genomes, which discards much of the nuclear genome. Recently, an alternative approach was proposed for sample identification, using unassembled genome skims, which can effectively improve phylogenetic signal and identification resolution. Studies have shown that the software Skmer and APPLES work well at estimating genomic distance and performing phylogenetic placement in birds and insects using low-coverage genome skims. In this study, we use Skmer and APPLES based on genome skims of 11 patellogastropods to perform assembly-free and alignment-free species identification and phylogenetic placement. Whether or not data corresponding to query species are present in the reference database, Skmer selects the best matching or closest species with COI barcodes under different sizes of genome skims except lacking species belonging to the same family as a query. APPLES cannot place patellogastropods in the correct phylogenetic position when the reference database is sparse. Our study represents the first attempt at assembly-free and alignment-free species identification of marine mollusks using genome skims, demonstrating its feasibility for patellogastropod species identification and flanking the necessity of establishing a database to share genome skims.

## 1. Introduction

The deterioration of the global ecosystems has accelerated biodiversity loss in recent years, including undiscovered germplasm resources. Rapid and inexpensive taxonomic identification to discover and protect biodiversity has become a hot topic among taxonomists. Currently, the standardized and broadly used method of species identification is DNA barcoding (e.g., COI, 12/16S, matK, ITS) [1,2,3,4], which is more efficient and accurate than previously widely used morphological identification methods. There is no doubt that DNA barcoding is the fundamental pillar of many current and future studies [5]. However, the high-quality DNA required for PCR amplification limits the available specimens, and the limited phylogenetic signals prevent barcoding from distinguishing all species (e.g., wasp) [6]. Moreover, although the Barcode of Life Data System (BOLD) (https://www.boldsystems.org/, accessed on 12 January 2022) provides a reliable database to assign an identity to query samples using a reference database of taxonomically preidentified vouchers, the barcode sequences contained in BOLD are only part of the biodiversity [7].

Recently, the decreasing costs of shotgun sequencing have led researchers to propose an alternative method, that is, using low-pass sequencing to generate genome skims [8,9], typically around 1–5 Gb [8,10], providing 0.1–10× coverage, and usually not enough to recover the nuclear genome [11]. Reconstructing organelle genomes from low-pass sequencing data to perform de novo reconstruction (e.g., maximum likelihood (ML) tree) is the most common application of genome skimming. There is no doubt that organelle genomes will provide a greater phylogenetic signal and identification resolution than barcoding in species identification [12,13,14,15]. However, the relatively time-consuming manual curation steps (i.e., assembly and alignment) and limited scalability prevent this method from being applied to ultra-large trees with higher accuracy and resolution. Furthermore, reconstructed phylogenetic relationships based on the mitochondrial genome are easily affected by long-branch attraction (LBA), which leads to the clustering of rapidly evolving taxa and increases the difficulty of reconstructing deep (Cambrian) molluscan evolution, especially with limited taxon sampling [16]. Most importantly, the above approach discards a vast proportion of the nuclear genome (as much as 99% of the sequence data) [5,17], making low-pass shotgun data underutilized.

The nuclear genome represents the evolutionary history of any nonclonal organism [5]. As the desirable approach to identifying samples, acquiring fully assembled nuclear genomes requires higher sequencing depth (at least 50×) and computational power [5,18]. In addition to the high sequencing cost and computing requirements, repeat elements of nuclear genomes will prevent unambiguous assembly when longer reads are sequenced. Although there are existing solutions to this problem (e.g., construction of mate-pair/large insert libraries for short-read technologies, high molecular weight DNA extraction, and long-read sequencing using single-molecule sequencing), the methods limit the available samples and require complex equipment and skills [5]. Therefore, a fully assembled nuclear genome is not presently suitable for extensive use in species identification.

Nevertheless, given that nuclear genome sequences represent the ultimate source of information for taxonomic assignment, some recent studies have proposed using all unassembled reads from low-pass sequencing data to perform taxonomic assignments [5,8,17]. It is argued that for a genome of size n and ignoring repeats, the probability of finding a k-mer with sufficient size (log_4_ n) in another genome relates directly to the evolutionary distance to the other genome [17]. Therefore, assembly-free and alignment-free species identification using genome skims might be a viable alternative to DNA barcoding. There is already some assembly-free or alignment-free software available [19,20,21], but this either requires high enough coverage (e.g., Co-phylog, Mash [21] and Simka) or the accuracy of the results is not high enough (e.g., AAF [20]). Recently, Sarmashghi et al. proposed a new assembly-free and alignment-free method for species identification and developed Skmer. This software can accurately compute the genomic distance from low-coverage genome skims [17]. Subsequently, Balaban et al. developed the software APPLES (Accurate Phylogenetic Placement using Least Squares) for distance-based phylogenetic placement, which can improve identification accuracy [22]. APPLES can find the optimal position for a new query species on an existing backbone (or reference) tree, which relies on the assembly-free estimates of genomic distance estimated from low coverage genome skims by Skmer or other alternatives. Compared to Maximum likelihood (ML) methods, APPLES is more memory efficient, more scalable, and runs faster.

Although both software performed well in the tests of birds and insects [17,22], broader testing is currently lacking. Patellogastropoda has been recognized as the most ‘primitive’ group of living gastropods. Due to historical vicariance and dispersal of the Pangean supercontinent, patellogastropod species with low dispersal ability have undergone geographic isolation and diverged on isolated islands, resulting in abundant cryptic species [23,24].

In this study, we sequenced the low-pass whole genome data of 11 patellogastropod species using genome skimming. Our aims were (1) to test the assembly-free and alignment-free species identification effectiveness of Skmer whether data correspond to query species in the reference database or not; (2) to test the phylogenetic placement effectiveness of APPLES in the above two cases; (3) to determine the minimum size of genome skims needed to obtain reliable species identification and phylogenetic placement results.

## 2. Material and Methods

### 2.1. DNA Extraction and Sequencing

The collecting site of each specimen is shown in Table 1. All specimens were immediately preserved in 95% ethanol following collection. The total genomic DNA was extracted using the TIANamp Marine Animals DNA Kit (TIANGEN Biotech Beijing Co., Ltd., Beijing, China), following the manufacturer’s protocols. Genomic DNA was sequenced by Novogene Technology Co., Ltd. (Beijing, China) on the Illumina NovaSeq 6000 platform using a PE150 protocol. To compare the species identification effectiveness of Skmer and APPLES between whether there is data corresponding to query species in the reference database or not, we selected *Scutellastra flexuosa*, *Cellana toreuma* (HN), and *Patelloida conulus* as the query species and other patellogastropod species constituted the reference library. *S. flexuosa* is the only species that belongs to Patellidae, while other families have at least three species in our study. *C. toreuma* (HN) and *C. toreuma* (GD) are specimens collected from different sampling sites. *P. conulus*, *Patelloida ryukyuensis,* and *Patelloida saccharina lanx* belong to the genus *Patelloida*. Query species from different scenarios allow us to better evaluate the application of assembly-free and alignment-free species identification using Skmer and APPLES in marine shellfish.

### 2.2. Genomic Reads Subsampling and Preprocessing

First, genome skims with 0.1 Gb, 0.5 Gb, 1 Gb, 2 Gb, and 4 Gb of data were generated from the paired-end reads using BBTools [25] by randomly subsampling the reads. As *L. goshimai* has the smallest size (7.3 Gb) of clean data in the study (Table S1), we used BBTools to subsample the paired-end reads of the remaining species to 7.3 Gb and called this the largest data in this article. Then we used fastp [26] to filter low-quality reads and remove adapters. Kraken II; [27] was used to filter possible extraneous reads such as bacteria, archaea, viral and human contamination. After filtering, BBtools was used to clean up deduplicate reads and merge paired-end reads. The statistics of the processing results for each step are shown in Table S3. Through this pipeline, we obtained the six datasets for the 11 patellogastropod species, and *Trochus maculatus* was selected as the outgroup.

### 2.3. Distance Calculation and Phylogenetic Placement

The process of Skmer calculation distance is as follows: First, Jelly Fish [28] was used to compute the frequency profiles of the subset of genome skims (i.e., 0.1 Gb, 0.5 Gb, 1 Gb, 2 Gb, 4 Gb, or largest data), and then to estimate the coverage, genome length, error rate, and read length, which can help analyze the test results further. Second, we used the hashing technique of Mash to retain a subset of the above subset of genome skims, and then it was used to compute the Jaccard index. Finally, we used these estimates to compute the genomic distance between the query and reference. To compare with the DNA barcoding method, COI barcodes for each species were downloaded from the BOLD database. MEGA v. 5.1 [29] was used to align all barcodes and calculate the pairwise *p*-distance.

After obtaining the distance matrix of reference samples, we transformed the genomic distances to Jukes–Cantor (JC) distances. Then, we used FastME [30] to infer the backbone tree, which is the necessary input for APPLES. Finally, APPLES used Treeswift [31] to place the query on the optimal position of the backbone tree based on the distance matrices mentioned above and used the JC69 model to independently compute phylogenetic distances [32] without the Γ model of rate variation for all pairs. Phylogenetic trees were visualized in FigTree v. 1.4.4 [33]. To further evaluate the effectiveness of the method, the reference mitogenome trees using exactly the same species and the outgroup were reconstructed based on 13 mitochondrial protein-coding genes (PCGs, refer to Xu et al. [34] for the specific method).

## 3. Results

**Figure 1:**
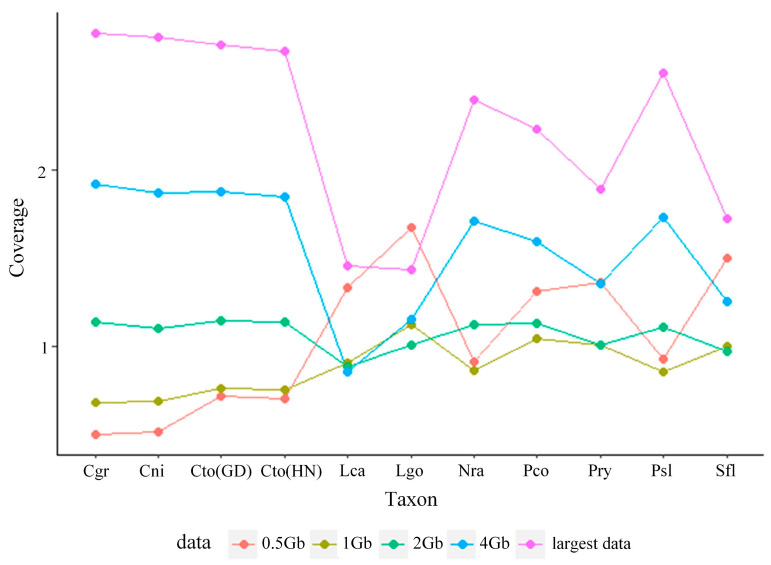
Coverage distribution of *P. saccharina lanx* (Psl), *P. conulus* (Pco), *P. ryukyuensis* (Pry), *C. toreuma* (GD) (Cto-GD), *C. toreuma* (HN) (Cto-HN), *C. grata* (Cgr), *C. nigrolineata* (Cni), *L. goshimai* (Lgo), *L. cassis* (Lca), *N. radula* (Nra), and *S. flexuosa* (Sfl) under different sizes of genome skims.

Our subsampled genome skims ranged in coverage from 0.50× to 1.68×, 0.69× to 1.13×, 0.63× to 1.15×, 0.69× to 1.92×, and 0.93× to 2.78× for 0.5 Gb, 1 Gb, 2 Gb, 4 Gb, and largest data, respectively (Figure 1; Table S2). However, Skmer was unable to estimate the coverage of 0.1 Gb data. We then compared estimated distances computed from COI and unassembled shotgun sequence data.

First, we select *C. toreuma* (HN) as the query species. Skmer correctly identified the best match under different sizes of genome skims with very small differences in the genomic distance except for 0.1 Gb (Table 2). Then, when *S. flexuosa* was used as the query species, the calculation results from different sizes of genome skims agreed that *P. saccharina lanx* was the closest species to the query skims in our reference samples. However, the pairwise distance of all COI barcodes calculated by MEGA v. 5.1 showed that *C. grata* and *C. toreuma* have a smaller genetic distance with *S. flexuosa* than *P. saccharina lanx* (Table 3). For *P. conulus*, both COI and genome skims agreed that the closest species was *P. ryukyuensis* (Table 4).

Although Nacellidae and Lottiidae were a monophyletic group and clustered into a clade in most backbone trees (Figures S1 and S2), subtle differences existed in the phylogenetic relationships of backbone trees inferred by FastME based on specific sizes of genome skims. Specifically, the backbone trees of 0.5 Gb and 1 Gb data placed Nacellidae as a paraphyletic grade at the base of patellogastropods in Figure S1. Nacellidae nested in the family Lottiidae in the backbone tree inferred based on 2 Gb genome skims. In Figure S2, Lottiidae nested within Nacellidae in the reference tree of 0.5 Gb. However, the backbone trees with the same phylogenetic relationship had different branch lengths due to different pairwise distances to query samples.

**Figure 2:**
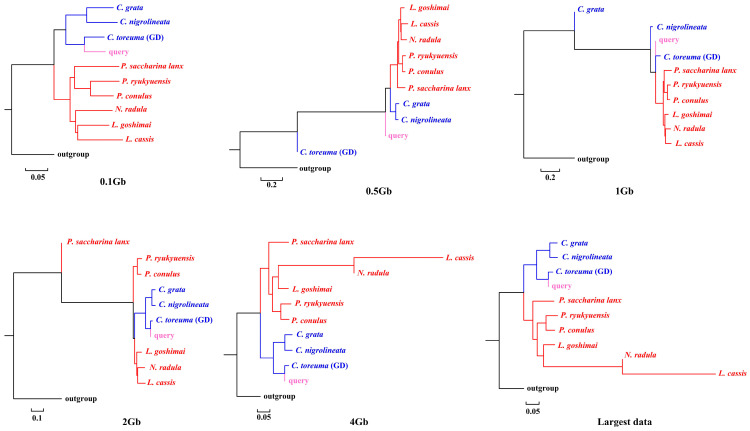
Phylogenetic placement of *C. toreuma* (query species) under different sizes of genome skims.

**Figure 3:**
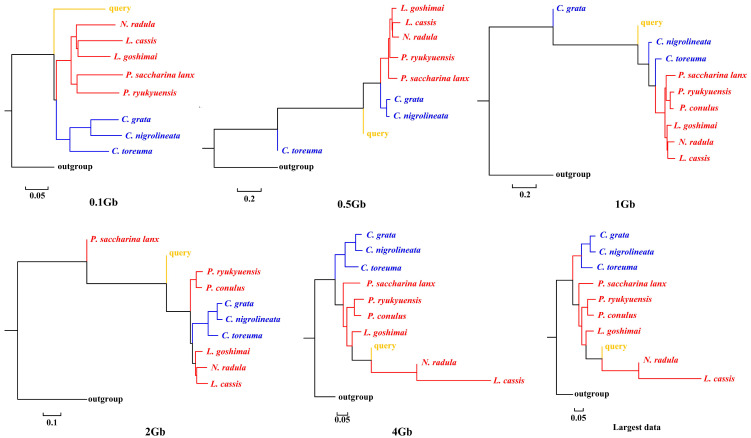
Phylogenetic placement of *S. flexuosa* (query species) under different sizes of genome skims.

**Figure 4:**
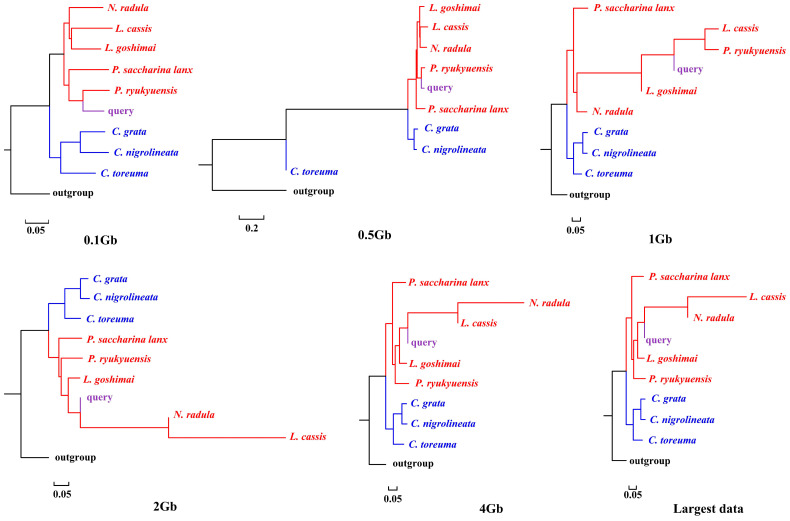
Phylogenetic placement of *P. conulus* (query species) under different sizes of genome skims.

APPLES placed *C. toreuma* (HN) into a clade with *C. toreuma* (GD) except for the 0.5 Gb data (Figure 2). The phylogenetic position of *S. flexuosa* was variable in the results obtained based on different sizes of genome skims (Figure 3). APPLES placed *S. flexuosa* at or near the base of Patellogastropoda except for the phylogenetic results of 4 Gb and the largest data, which placed the query into a sister clade with *L. cassis* and *N. radula*. When *P. conulus* was selected as the query species, it formed a clade with *P. ryukyuensis* or *P. ryukyuensis* + *L. cassis* under 0.1 Gb, 0.5 Gb, and 1 Gb data, while it was sister to a clade formed by *N. radula* and *L. cassis* in the placement results based on data volumes of 2 Gb and above (Figure 4).

## 4. Discussion

We present here the first assembly-free and alignment-free species identification using genome skims on Patellogastropoda. Skmer consistently selected the best matching or closest species under different data amounts, whether there were data corresponding to the query in the reference database or not. However, when species belonging to the same family were lacking, the taxon selection of COI barcodes was different from that of genome skims. APPLES placed query species in the accurate phylogenetic position only when the corresponding species data were available in the reference database.

### 4.1. Coverage

In our study, the coverage of genome skims estimated by Skmer was less than 3×, even in the largest data. The coverage was not uniform; it was randomly distributed and did not increase exponentially as the genome skims increased, which may be due to the overrepresentation of mitochondrial sequences (Figure 1, Table S2) [17].

### 4.2. Selection of the Best Matched Species

In our study, Skmer selected the same species that best matched the query sequence in the reference datasets even based on different sizes of genome skims. For *C. toreuma* (HN), Skmer selected the correct best matching species. The closest species of *P. conulus* selected based on different sizes of genome skims and COI was consistent. Interestingly, *P. saccharina lanx*, which belongs to Lottiidae, was selected by Skmer as the best matching species for *S. flexuosa*, but the result of COI barcodes showed that *C. grata* and *C. toreuma* had a much closer genomics distance with *S. flexuosa* than *P. saccharina lanx*.

According to the estimated distances, we found no correlation between the results of COI and genome skims even though both of them selected the same best matching species (Table 2, Table 3 and Table 4). There are several possible reasons for this discrepancy between COI barcoding and genome skims. First, the length of COI barcoding is around 658 bp, which can only provide limited sequence information and low phylogenetic resolution [35]. In contrast, genome skims not only include all of the different ‘standard’ animal barcoding regions (e.g., COI, 12S, 16S), but also provide sequence data from other loci [8,36], which hold valuable information that can further achieve the goals of species identification. In genome skimming approaches, as much as 99% of the sequence data is from the nuclear genome [5,17,37]. Considering this fact, we speculate that the second reason could be the great difference in sequence information provided between organelle and nuclear genome, which may result in gene tree/species tree discordances. For example, Patellogastropoda and Vetigastropoda were recovered as the sister clade of the remaining gastropods based on transcriptomes [38], while Patellogastropoda was the sister lineage of the remaining gastropods when reconstructed based on the mitochondrial genome [16]. The third possible reason is that Patellogastropoda might have complex mutations, such as large-scale repetition, especially *S. flexuosa*. When calculating distance based on genome skims, Skmer will simplify the evolutionary process, such as ignoring repeats and assuming that mutations are uniformly distributed [5,17], which might reduce the accuracy of the results of the calculation about the patellogastropods. While in the calculation with COI barcodes, the *p*-distance or Kimura 2-parameter model is usually chosen according to the affinity between the query species and the reference species. The potential inherent biases in the methods may also explain the observed differences between COI and genome skims.

In addition, a crucial step before the application of assembly-free and alignment-free species identification is to use Kraken-II to remove possible contamination reads after subsampling. Several facts about Kraken-II might affect the accuracy of calculated distance. Firstly, studies have shown that Kraken-II can effectively reduce the adverse effects of contamination only when the contaminants and the contaminant reference library have a match, which is within a 5–10% genomic distance [5,11]. Therefore, an incomplete contaminant reference library and unconfident matching to the contaminant database may affect our estimation results. Secondly, impure query or reference skims can lead to underestimating the accurate distance by Skmer, especially when the impurity of the query skims is similar to that of the reference skims [5,11]. Thirdly, Kraken-II has been shown to occasionally over-correct errors, which might lead to an overestimation of the true distance [11].

### 4.3. Phylogenetic Placement

According to the phylogenetic placement result, APPLES can accurately determine the location of the query species above 1 Gb genome skims when the corresponding species data are available in the reference database. Although stable phylogenetic positions were obtained based on 4 Gb and above data, the results were irrational when the reference database did not have corresponding query data. The phylogenetic tree reconstructed by mitogenomes (Figure S3; [34]), mitochondrial and nuclear genes [23] showed that *P. conulus* has a closer relationship with *P. ryukyuensis*. However, APPLES placed it in the sister clade of *N. radula* and *L. cassis*. *S. flexuosa,* which lacks species belonging to the Patellidae family in the backbone tree, was nested in Lottiidae and then formed a clade with *N. radula* and *L. cassis.* However, in the phylogenetic reconstruction based on the mitochondrial genome, *S. flexuosa* was clustered into a clade with Nacellidae (Figure S4; [34]) and was placed in the most basal position in the molecular phylogeny, including the most extensive sampling of specimens [23]. The results indicated that APPLES could not place *P. conulus* and *S. flexuosa* in the exact phylogenetic position.

In addition to the possible influencing factors mentioned above in the Skmer section, we considered that the misplacement of *P. conulus* and *S. flexuosa* might also be influenced by sparse taxon sampling, which might decrease the accuracy of APPLES. Similarly, in the phylogenetic inference of mitochondrial genomes, the evolutionary relationship of *L. digitalis* suffers from long-branch attraction (LBA), which results in inconsistent relationships among different studies [16,39]. Nevertheless, it has recently been proven that improved taxon sampling can effectively alleviate the LBA [34]. Future studies using a denser taxon sampling, especially in *Patelloida* and Patellidae, can further confirm our hypothesis.

The above evidence shows that APPLES cannot place patellogastropods in a reliable phylogenetic position on a sparse backbone tree.

## 5. Conclusions

In our study, the assembly-free and alignment-free methods for species identification using genome skims perform well in Patellogastropoda, meaning that Skmer has good potential for application in more taxa besides insects and birds. Problems that existed in the phylogenetic placement of APPLES might be affected by limited taxon sampling and need to be further discussed by increasing the number of species in *Patelloida* and Patellidae. More comparative studies covering denser sampling and different groups of mollusks should be implemented in the future. Furthermore, to better apply this new species identification method, we propose establishing a large reference database to store the processed shotgun sequencing data.

## Figures and Tables

**Table 1:** List of species used in this study.

Subclass	Family	Species	Locality
Patellogastropoda			
	Nacellidae		
		*Cellana toreuma*	Yangjiang, Guangdong, China
			Wenchang, Hainan, China
		*Cellana nigrolineata*	Jeju Island, South Korea
		*Cellana grata*	Ningde, Fujian, China
	Patellidae		
		*Scutellastra flexuosa*	Sansha, Hainan, China
	Lottiidae		
		*Nipponacmea radula*	Weihai, Shandong, China
		*Patelloida ryukyuensis*	Weihai, Shandong, China
		*Patelloida saccharina lanx*	Wenchang, Hainan, China
		*Patelloida conulus*	Weihai, Shandong, China
		*Lottia cassis*	Weihai, Shandong, China
		*Lottia goshimai*	Qingdao, Shandong, China
Vetigastropoda			
	Trochidae		
		*Trochus maculatus*	Sanya, Hainan, China

**Table 2:** The calculation distance from COI and different sizes of genome skims between *C. toreuma* (HN) and reference species in our study. Color shows the distance ranking between reference species and query species, that is, the darker the color, the farther the relationship.

	COI	0.1 Gb	0.5 Gb	1 Gb	2 Gb	4 Gb	Largest Data
*C. toreuma* (GD)	0.003	0.0918	0.0095	0.0115	0.0131	0.0145	0.0159
*C. grata*	0.174	0.1981	0.1272	0.1334	0.1359	0.1382	0.1406
*C. nigrolineata*	0.187	0.1987	0.1236	0.1299	0.1337	0.1373	0.1397
*P. ryukyuensis*	0.368	0.2727	0.1986	0.2261	0.2318	0.2531	0.2554
*P. conulus*	0.384	0.2630	0.1985	0.2106	0.2254	0.2395	0.2410
*L. goshimai*	0.392	0.2541	0.1883	0.1955	0.2042	0.2174	0.2229
*P. saccharina lanx*	0.399	0.2480	0.1807	0.2041	0.2112	0.2230	0.228
*L. cassis*	0.676	0.2705	0.2078	0.2311	0.2342	0.2479	0.2609
*N. radula*	0.684	0.2439	0.2016	0.2112	0.2116	0.2320	0.2389

Color from light to dark: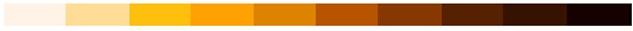.

**Table 3:** The calculation distance from COI and different sizes of genome skims between *S. flexuosa* and reference species in our study. Color shows the distance ranking between reference species and query species, that is, the darker the color, the farther the relationship.

	COI	0.1 Gb	0.5 Gb	1 Gb	2 Gb	4 Gb	Largest Data
*C. grata*	0.216	0.2541	0.1953	0.2138	0.2174	0.2359	0.2408
*C. toreuma*	0.216	0.2439	0.1697	0.1806	0.1974	0.2139	0.2230
*C. nigrolineata*	0.224	0.2582	0.1926	0.2077	0.2173	0.2365	0.2470
*L. goshimai*	0.376	0.2503	0.1806	0.1850	0.2037	0.2179	0.2229
*P. ryukyuensis*	0.379	0.2705	0.1905	0.2120	0.2194	0.2384	0.2379
*P. conulus*	0.388	0.2480	0.1885	0.2011	0.2112	0.2218	0.2299
*P. saccharina lanx*	0.408	0.2330	0.1637	0.1788	0.1881	0.2003	0.2043
*L. cassis*	0.654	0.2727	0.1961	0.2141	0.2407	0.2403	0.2526
*N. radula*	0.673	0.2480	0.1863	0.2030	0.2217	0.2317	0.2360

Color from light to dark: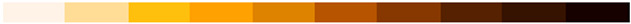.

**Table 4:** The calculation distance from COI and different sizes of genome skims between *P. conulus* and reference species in our study. Color shows the distance ranking between reference species and query species, that is, the darker the color, the farther the relationship.

	COI	0.1 Gb	0.5 Gb	1 Gb	2 Gb	4 Gb	Largest Data
*P. ryukyuensis*	0.152	0.1155	0.0501	0.0570	0.0656	0.0729	0.0778
*P. saccharina lanx*	0.234	0.1900	0.1308	0.1375	0.1481	0.1581	0.1627
*L. goshimai*	0.245	0.1744	0.1044	0.1122	0.1216	0.1263	0.1288
*L. cassis*	0.306	0.2176	0.152	0.1574	0.1666	0.1787	0.1797
*N. radula*	0.325	0.1951	0.1310	0.1434	0.1528	0.1597	0.1614
*C. nigrolineata*	0.359	0.2775	0.2353	0.2434	0.2394	0.2458	0.2524
*C. grata*	0.362	0.2802	0.2225	0.2487	0.2445	0.2435	0.2501
*C. toreuma*	0.365	0.2630	0.1986	0.2041	0.2300	0.2491	0.2467

Color from light to dark: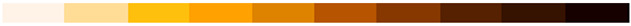.

## Data Availability

The original shotgun sequencing data used in this study were deposited in the NCBI Sequence Read Archive (BioProject PRJNA766309, accessed on 7 June 2022).

## Supplementary Materials

The following supporting information can be downloaded at: https://www.mdpi.com/article/10.3390/genes13071192/s1, Figure S1: The backbone tree of *C. toreuma* (HN) and *S. flexuosa* inferred by FastME under different size of genome skims; Figure S2: The backbone tree of *P. conulus* inferred by FastME under different size of genome skims; Figure S3: Phylogenetic relationships of *P. conulus* (query species) inferred based on concatenated amino acids of 13 mitochondrial protein-coding genes. Numbers at nodes are statistical support values for BI (posterior probabilities)/ML (bootstrap proportions in percentage); Figure S4: Phylogenetic relationships of *S. flexuosa* (query species) inferred based on concatenated amino acids of 13 mitochondrial protein-coding genes. Numbers at nodes are statistical support values for BI (posterior probabilities)/ML (bootstrap proportions in percentage); Table S1: The original paired-end reads; Table S2: The parameters of different size of genome skims calculated by Skmer; Table S3: The statistics of the processing results for BBTools, fastp and Kraken II.
