# Supplementary material for: Testing Efficacy of Assembly-Free and Alignment-Free Methods for Species Identification Using Genome Skims, with Patellogastropoda as a Test Case
Source: Genes (Basel). 2022 Jul 2;13(7):1192. doi: 10.3390/genes13071192 (PMC9318368; doi:10.3390/genes13071192)
Supplement: Supplementary file 1 [file genes-13-01192-s001.zip › Table S1-S2; Figure S1-S4.pdf]

Table S1. The original paired-end reads.

Species	Uncompressed file/kb
L. cassis	10,091,509/10,091,509
N. radula	7,467,310/7,467,310
C. grata	8,553,973/8,553,973
L. goshimai	7,454,544/7,454,544
P. conulus	14,760,357/14,760,357
P. saccharina lanx	14,461,217/14,461,217
P. ryukyuensis	16,165,902/16,165,902
C. nigrolineata	12,972,845/12,972,845
C. toreuma (GD)	14,172,310/14,172,310
C. toreuma (HN)	18,663,524/18,663,524
S. flexuosa	12,406,821/12,406,821

Table S2. The parameters of different size of genome skims calculated by Skmer.

0.1G

	Coverage	Genome_length	Error_rate	Read_length
S. flexuosa	NA	NA	NA	229
L. cassis	NA	NA	NA	235
N. radula	NA	NA	NA	234
C. grata	NA	NA	NA	238
L. goshimai	NA	NA	NA	234
P. conulus	NA	NA	NA	233
P. saccharina lanx	NA	NA	NA	232
P. ryukyuensis	NA	NA	NA	234
C. nigrolineata	NA	NA	NA	243
C. toreuma (GD)	NA	NA	NA	237
C. toreuma (HN)	NA	NA	NA	234

0.5G

	Coverage	Genome_length	Error_rate	Read_length
S. flexuosa	1.5013287792624153	95750346	0.028200929350726822	229
L. cassis	1.3327740387547855	45463855	0.027914606109592177	234
N. radula	0.9152781068328262	110192677	0.01567515129507513	234
C. grata	0.5036046394650663	174661355	0.008291417727817385	238
L. goshimai	1.6776567653334804	49808401	0.028761549753830717	234
P. conulus	1.314254793107782	102469388	0.02150057228294544	233
P. saccharina lanx	0.9278481344981513	119876574	0.01775294465663002	232
P. ryukyuensis	1.3659537009334826	75619675	0.02491465125299086	234
C. nigrolineata	0.5179819107059459	148918377	0.010116997073165712	243
C. toreuma (GD)	0.7217787063366554	144208905	0.013825683369604325	237
C. toreuma (HN)	0.7051828916055268	144004463	0.01409483746843987	234

1G

	Coverage	Genome_length	Error_rate	Read_length
S. flexuosa	1.0029473619968219	280995598	0.016122217186681498	229
L. cassis	0.9050214990722926	129731681	0.015727142054512533	234
N. radula	0.8636988451143478	226908173	0.007429620144492	234
C. grata	0.6852204637580797	251596063	0.0037109939709843998	238
L. goshimai	1.1259212744418114	143346414	0.017574206420095217	234
P. conulus	1.0438850315377552	253264561	0.012019595240989833	233
P. saccharina lanx	0.8560015672871802	253444900	0.008890363121627054	232
P. ryukyuensis	1.0079677742963102	200437899	0.014262985885386215	234
C. nigrolineata	0.6892807532643411	220849531	0.005215118095972748	243
C. toreuma (GD)	0.7659604249090144	268961901	0.006168678299533492	237
C. toreuma (HN)	0.758536873487782	264678395	0.006597347393543118	234

2G

	Coverage	Genome_length	Error_rate	Read_length
S. flexuosa	0.9739603350231031	568184167	0.008350984478455836	229
L. cassis	0.8836878220845178	254302203	0.007915517638578007	234
N. radula	1.1234930557432787	333400647	0.0033993059939037007	234
C. grata	1.1398227918679955	291051615	0.0021502725881005214	238
L. goshimai	1.0073525901651545	308002573	0.009496062842218045	234
P. conulus	1.1328283557791097	460810310	0.006000928817245121	233
P. saccharina lanx	1.1087942016526018	382399050	0.00439628172456763	232
P. ryukyuensis	1.012443020367157	393579569	0.00699520386915653	234
C. nigrolineata	1.105576596622458	272373415	0.0029954150060985674	243
C. toreuma (GD)	1.149406241031329	355042049	0.0032381573580544387	237
C. toreuma (HN)	1.1362944576862781	350783337	0.0035828860869960844	234

4G

	Coverage	Genome_length	Error_rate	Read_length
S. flexuosa	1.255199716467791	843502458	0.004405105731694392	229
L. cassis	0.8546704594611217	205411753	0.010290676984266778	234
N. radula	1.7091781542083455	400260113	0.001975268301126487	234
C. grata	1.9215991714931617	316407172	0.0015958020516907423	238
L. goshimai	1.1533818579078017	489368197	0.004877242961904393	233
P. conulus	1.5924890918985637	630852603	0.00342944974093351	233
P. saccharina lanx	1.7314412846506766	467909490	0.0027453164655780737	232
P. ryukyuensis	1.353249417848962	566191664	0.003609606512741781	233
C. nigrolineata	1.8734610083546972	309937954	0.002118300972162257	243
C. toreuma (GD)	1.8795930711427973	419087036	0.0020809347574954895	237
C. toreuma (HN)	1.845035501662378	417354301	0.0023113595691213185	234

largest data

	Coverage	Genome_length	Error_rate	Read_length
S. flexuosa	1.728193678897708	1019697975	0.0030005034435302003	229
L. cassis	1.4576399556336794	437398847	0.002521865597942541	234
N. radula	2.394840588569781	446504425	0.0015236807962926147	234
C. grata	2.7773452574667132	340994281	0.0014036933986266709	238
L. goshimai	1.4395252523196236	605976119	0.00313680064894728	233
P. conulus	2.2315570578672017	753834277	0.00257679334746419	233
P. saccharina lanx	2.5478325003695264	528630228	0.0022658388657428308	232
P. ryukyuensis	1.8917555040703409	677175949	0.002434969677229337	233
C. nigrolineata	2.7512881584743245	352478197	0.0017901738425651903	243
C. toreuma (GD)	2.709253588460741	486739748	0.0016482778811850451	237
C. toreuma (HN)	2.6691480178728657	484473177	0.0018889276975615488	234

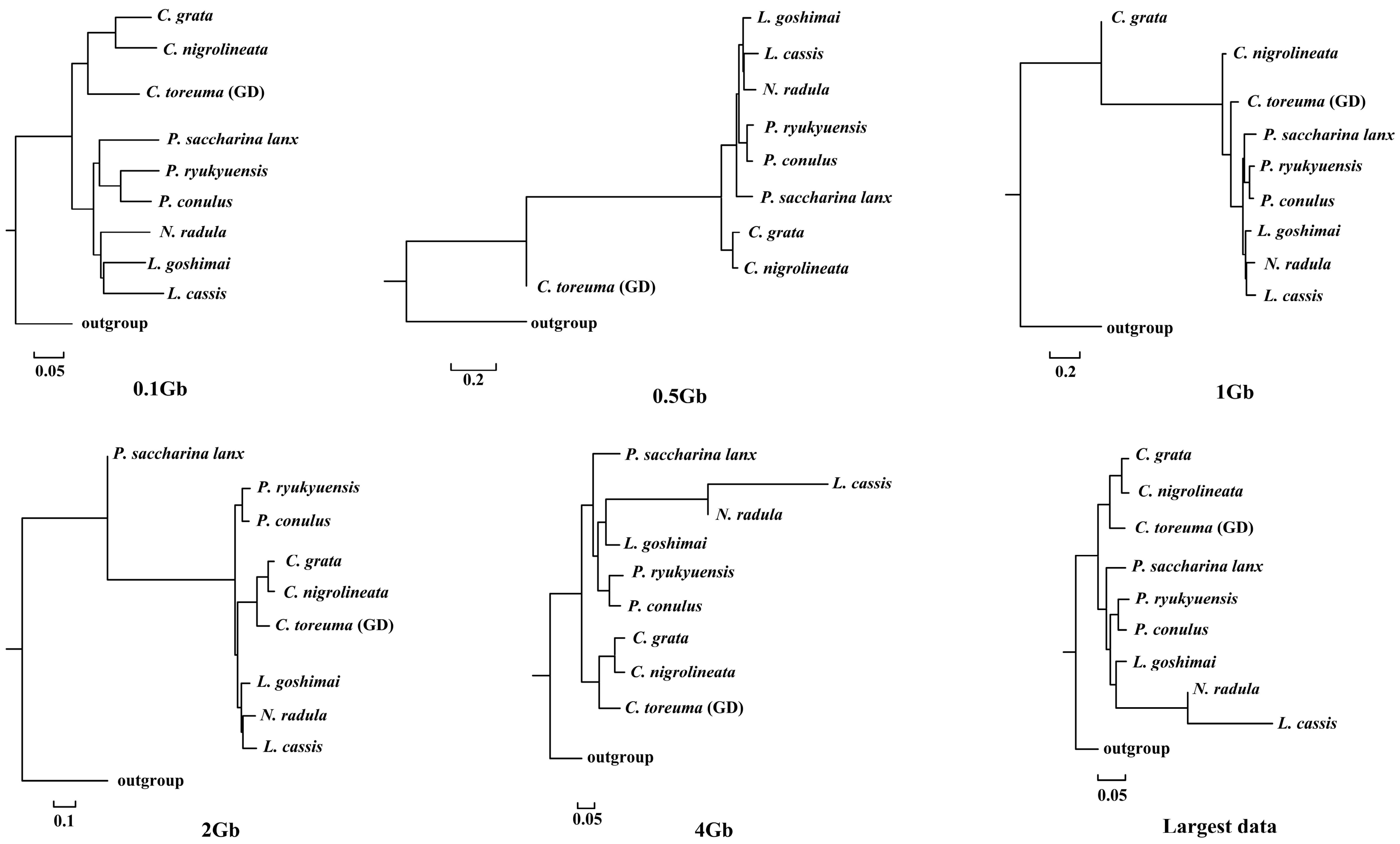

Figure S1. The backbone tree of *C. toreuma* (HN) and *S. flexuosa* inferred by FastME under different size of genome skims.

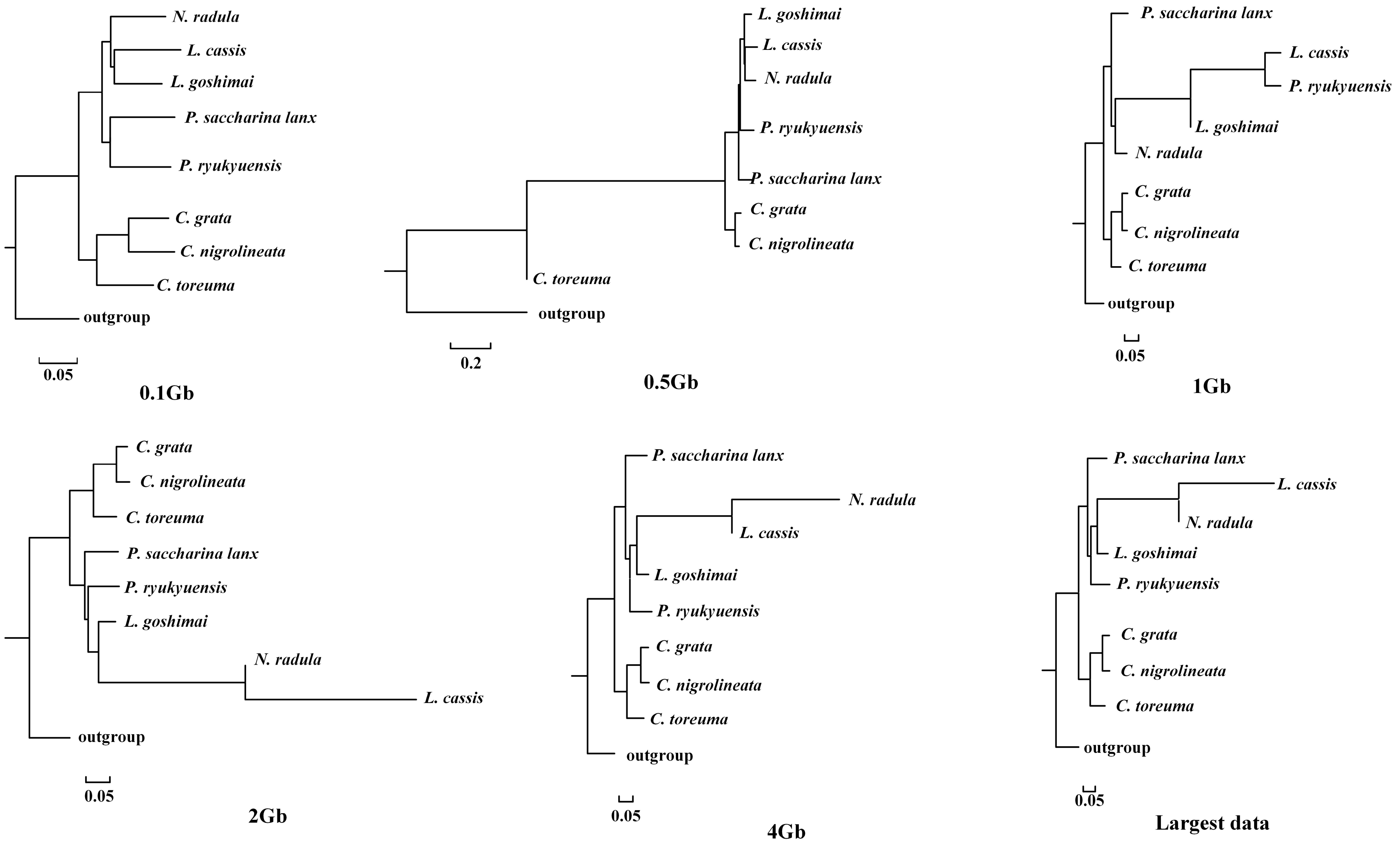

N. radula
L. cassis
L. goshimai
P. saccharina lanx
P. ryukyuensis
C. grata
C. nigrolineata
C. toreuma
outgroup
0.05
0.1Gb
L. goshimai
L. cassis
N. radula
P. ryukyuensis
P. saccharina lanx
C. grata
C. nigrolineata
C. toreuma
outgroup
0.2
0.5Gb
P. saccharina lanx
L. cassis
P. ryukyuensis
L. goshimai
N. radula
C. grata
C. nigrolineata
C. toreuma
outgroup
0.05
1Gb
C. grata
C. nigrolineata
C. toreuma
P. saccharina lanx
P. ryukyuensis
L. goshimai
N. radula
L. cassis
outgroup
0.05
2Gb
P. saccharina lanx
N. radula
L. cassis
L. goshimai
P. ryukyuensis
C. grata
C. nigrolineata
C. toreuma
outgroup
0.05
4Gb
P. saccharina lanx
L. cassis
N. radula
L. goshimai
P. ryukyuensis
C. grata
C. nigrolineata
C. toreuma
outgroup
0.05
Largest data

Figure S2. The backbone tree of *P. conulus* inferred by FastME under different size of genome skims.

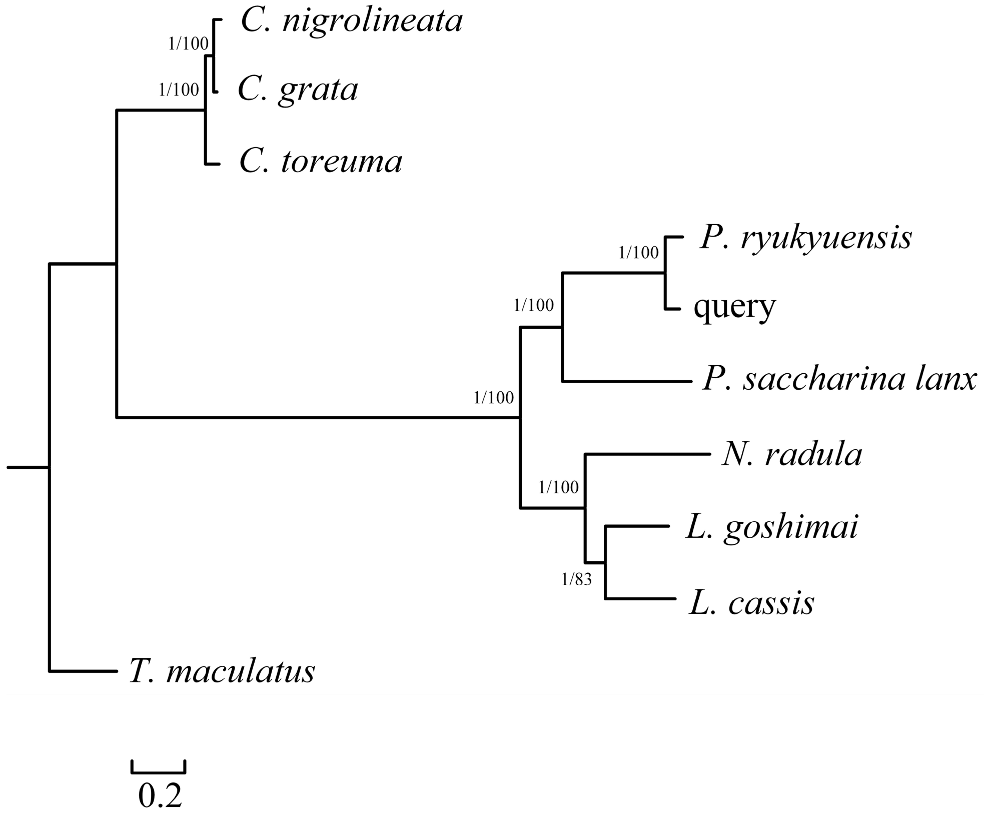

Figure S3. Phylogenetic relationships of *P. conulus* (query species) inferred based on concatenated amino acids of 13 mitochondrial protein-coding genes. Numbers at nodes are statistical support values for BI (posterior probabilities)/ML (bootstrap proportions in percentage).

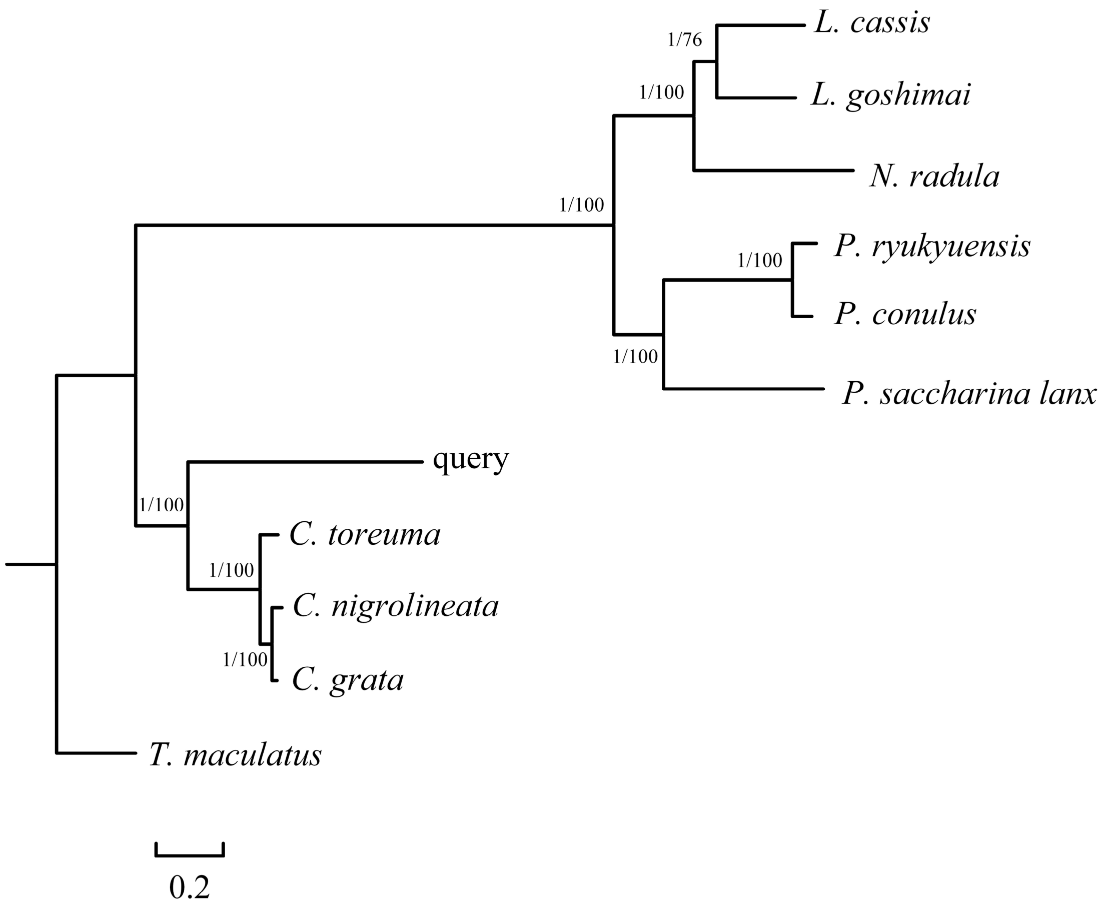

Figure S4. Phylogenetic relationships of *S. flexuosa* (query species) inferred based on concatenated amino acids of 13 mitochondrial protein-coding genes. Numbers at nodes are statistical support values for BI (posterior probabilities)/ML (bootstrap proportions in percentage).
